# Dynamic aerosol and dynamic air-water interface curvature effects on a 2-Gbit/s free-space optical link using orbital-angular-momentum multiplexing

**DOI:** 10.1515/nanoph-2021-0516

**Published:** 2021-11-04

**Authors:** Haoqian Song, Runzhou Zhang, Nanzhe Hu, Huibin Zhou, Xinzhou Su, Hao Song, Kaiheng Zou, Kai Pang, Cong Liu, Daeyoung Park, Brittany Lynn, Greg Gbur, Aristide Dogariu, Richard J. Watkins, Jerome K. Miller, Eric Johnson, Moshe Tur, Alan E. Willner

**Affiliations:** University of Southern California, Los Angeles 90089, CA, USA; INHA University, Incheon 22212, South Korea; Naval Information Warfare Center Pacific, San Diego, CA 92152, USA; University of North Carolina at Charlotte, Charlotte, NC 28223, USA; University of Central Florida, Orlando, FL 32816, USA; Clemson University, Clemson, SC 29634, USA; Tel Aviv University, Ramat Aviv 69978, Israel

**Keywords:** air–water interface, free-space optical communication, mode division multiplexing, orbital angular momentum mode

## Abstract

When an orbital-angular-momentum (OAM) beam propagates through the dynamic air–water interface, the aerosol above the water and the water surface curvature could induce various degradations (e.g., wavefront distortion, beam wandering, scattering, and absorption). Such time-varying degradations could affect the received intensity and phase profiles of the OAM beams, resulting in dynamic modal power loss and modal power coupling. We experimentally investigate the degradation for a single OAM beam under dynamic aerosol, dynamic curvature, and their comprehensive effects. Our results show the following: (i) with the increase of the aerosol strength (characterized by the attenuation coefficient) from ∼0 to ∼0.7–1.3 dB/cm over ∼7 cm, the power coupling ratio from OAM −1 to +2 increases by 4 dB, which might be due to the amplitude and phase distortion caused by spatially dependent scattering and absorption. (ii) With the increase of the curvature strength (characterized by the variance of curvature slope over time) from ∼0 to ∼2 × 10^−5^ rad^2^, the power coupling ratio from OAM −1 to +2 increases by 11 dB. This could be caused by both the wavefront distortion and the beam wandering. (iii) Under the comprehensive effect of aerosol (∼0.1–0.6 dB/cm) and curvature (∼6 × 10^−7^ rad^2^), there is an up to 2 dB higher modal power loss as compared with the single-effect cases. (iv) The received power on OAM −1 fluctuates in a range of ∼6 dB within a 220 ms measurement time under aerosol (∼0.1–0.6 dB/cm) and curvature (∼6 × 10^−7^ rad^2^) effects due to the dynamic degradations. We also demonstrate an OAM −1 and +2 multiplexed 2-Gbit/s on–off-keying link under dynamic aerosol and curvature effects. The results show a power penalty of ∼3 dB for the bit-error-rate at the 7% forward-error-correction limit under the comprehensive effect of aerosol (∼0.1–0.6 dB/cm) and curvature (∼6 × 10^−7^ rad^2^), compared with the no-effect case.

## 1 Introduction

Free-space optical communications has gained much interest due to its potential for higher capacity and lower probability of interception, as compared to radio-frequency techniques [1], [2], [3]. Moreover, using optical communications for underwater links has also gained attention for similar reasons, especially when compared to acoustic techniques [4], [5], [6]. Whereas links through the air can be performed in the visible or near-infrared (IR) region, underwater links tend to be in the blue-green region to minimize water absorption induced optical losses [4], [5], [6].

An interesting scenario is the transmission of data between a transceiver above the water and one below the water, such as links between a drone/plane and an underwater sensor/submarine [7], [8], [9]. In this case, the optical beam would pass through a fairly dynamic, complex, and potentially harsh air–water interface, that can have dynamic aerosol above the water and dynamic curvature of the water surface [10], [11], [12]. Previous reports have investigated the aerosol and curvature effects for a typical Gaussian beam: (i) the aerosol effect could lead to scattering and absorption of the beam [12], and (ii) the curvature effect could induce beam wandering and wavefront distortion [13], [14], [15], [16]. In addition, the comprehensive effects of aerosol and curvature have also been investigated for a beam transmitting through the air–water interface [12, 17]. These separate and comprehensive effects could result in system performance degradation (e.g., power loss) for a Gaussian-beam-based FSO link [12], [13], [14], [15].

These effects of the air–water interface can be further complicated when there are multiple optical beams with unique amplitude and phase profiles instead of a single fundamental Gaussian beam. One example is the orbital-angular-momentum (OAM) beams, which is a subset of Laguerre–Gaussian (LG) modes [18]. An OAM beam has: (i) a wavefront that “twists” as it propagates, (ii) an order of *l* which is the number of 2π phase shifts in the azimuthal direction, and (iii) an intensity profile that has a “donut” shape [19]. OAM beams with different orders are mutually orthogonal, which enables efficient multiplexing, coaxial-propagation, and demultiplexing of multiple OAM beams with little inherent crosstalk [20], [21], [22], [23]. For an OAM beam, the distortion of the beam’s unique intensity and phase profile could lead to power loss of the transmitted mode and power coupling to the neighboring modes [24]. Such degradations could induce both power loss and channel crosstalk in OAM multiplexed links, where multiple OAM beams are simultaneously transmitted, each carrying an independent data channel [24]. There have been reports of transmitting OAM beams through dynamic aerosol without carrying data [25], [26], [27] or transmitting a single data-carrying OAM beam through a relatively flat air–water interface [28]; however, little has been reported on the transmission of OAM modes when the comprehensive effects of aerosol and curvature are dynamically present, nor on the OAM multiplexing under aerosol and/or curvature effects.

In this paper, we experimentally investigate the dynamic aerosol and dynamic water surface curvature effects on the OAM beam transmission, and we demonstrate an air-to-water 2-Gbit/s on–off keying (OOK) OAM multiplexed FSO communication link [29, 30]. The detrimental effects of the dynamic aerosol and curvature on the transmitted OAM beams include but are not limited to wavefront distortion, beam wandering, scattering, and absorption. Such time-varying degradation could affect the received intensity and phase profiles of OAM beams, resulting in the dynamic power loss of the transmitted mode and power coupling to the neighboring modes. In this experiment, we generate the aerosol and curvature effects in a laboratory environment by using a vaporizer and a wind generator, respectively. Moreover, we characterize the two aerosol cases by their attenuation coefficients (∼0.1–0.6 and ∼0.7–1.3 dB/cm for aerosol cases 1 and 2, respectively, over ∼7 cm), and characterize the two curvature cases by their variances of the curvature slope over time (∼6 × 10^−7^ and ∼2 × 10^−5^ rad^2^ for curvature cases 1 and 2, respectively, with a maximum wave height of several millimeters). Our results show the following: (i) with the increase of the aerosol strength from no-aerosol to aerosol cases 1 and 2, the power coupling ratio from OAM −1 to +2 increases by 2 and 4 dB, respectively. This might be due to the amplitude and phase distortion induced by spatially dependent scattering and absorption. (ii) The scattering and absorption of aerosol also induce a power loss of the transmitted beam for >1 and >5 dB under aerosol cases 1 and 2, respectively. (iii) With the increase of the curvature strength from no-curvature to curvature cases 1 and 2, the power coupling ratio from OAM −1 to +2 increases by 3 and 11 dB, respectively. This could be caused by both the wavefront distortion and the beam wandering. (iv) Under the comprehensive effect of aerosol (∼0.1–0.6 dB/cm) and curvature (∼6 × 10^−7^ rad^2^), there is an up to 2 dB higher modal power loss as compared with the single-effect cases. (v) The received power on OAM −1 fluctuates in a range of ∼6 dB within a 220 ms measurement time under the aerosol (∼0.1–0.6 dB/cm) and curvature (∼6 × 10^−7^ rad^2^) effects due to the dynamic degradations. We also demonstrate OAM −1 and +2 multiplexed 2-Gbit/s OOK links under the dynamic aerosol and interface curvature. The results show a power penalty of ∼3 dB for the bit-error-rate (BER) at the 7% forward error correction (FEC) limit under the comprehensive effect of aerosol (∼0.1–0.6 dB/cm) and curvature (∼6 × 10^−7^ rad^2^), when compared with the no-effect case.

## 2 Concept

**Figure 1:**
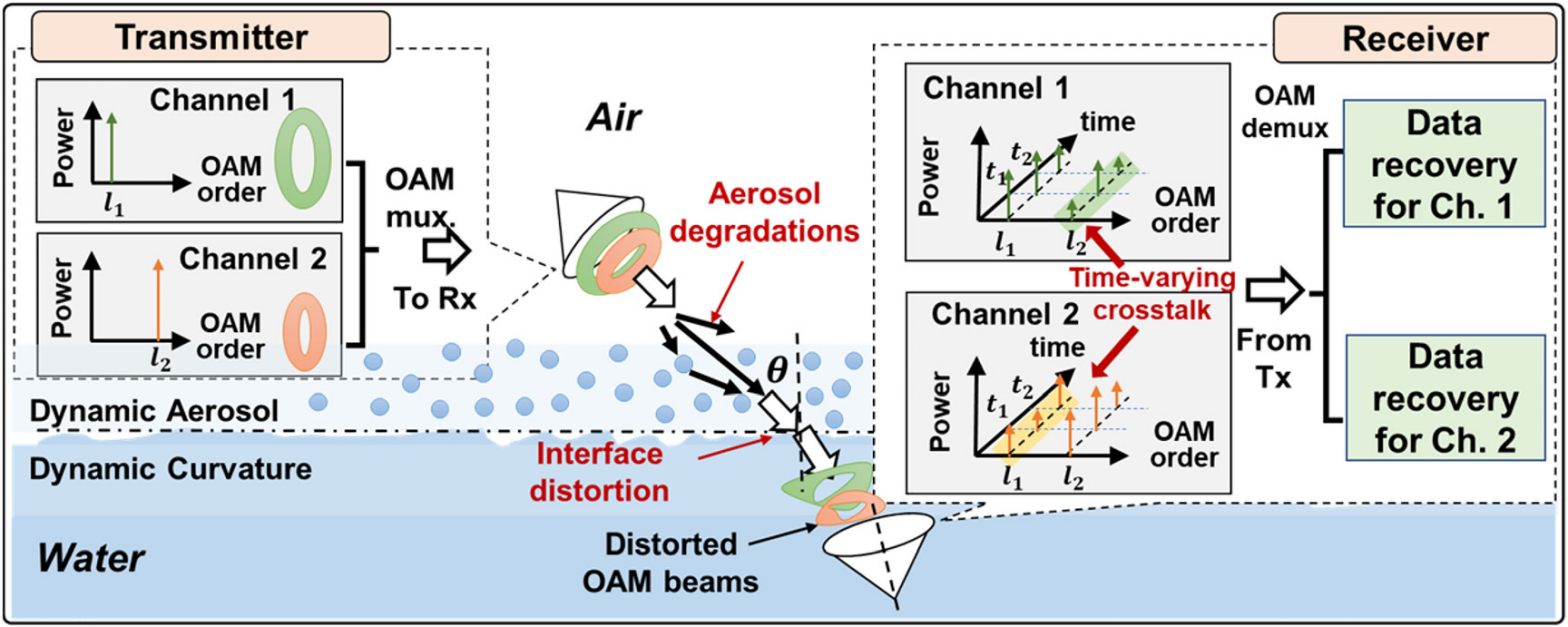
Concept of the OAM multiplexed link through the dynamic air–water interface. The dynamic aerosol and water surface curvature would induce time-varying power loss of the transmitted mode and crosstalk to neighboring modes. *θ*, angle of incidence; Tx, transmitter; Rx, receiver; Ch, channel.

There is growing interest in using structured light beams for FSO communications in atmospheric and underwater environments [31], [32], [33], [34], [35]. In this paper, we investigate the degradation of FSO links using OAM beams (i.e., a subset of spatially structured beams) through dynamic aerosol and curvature of the air–water interface, as shown in Figure 1. Each independent data channel is carried by a unique OAM beam, and multiple data-carrying OAM beams are multiplexed and coaxially propagate toward the air–water interface with an angle of incidence *θ*. At the dynamic air–water interface, the OAM beams could be degraded by the interface effects, including but not limited to dynamic aerosol and dynamic water surface curvature. Specifically, the curvature effect could potentially induce beam wandering and wavefront distortion [13], [14], [15], [16], and the inhomogeneous aerosol effect could induce spatially dependent scattering and attenuation toward the beam [12, 25]. When recovering the OAM beams at the receiver, there would be (i) power loss of the transmitted modes, which would reduce the signal-to-noise ratio (SNR) of the recovered signal, and (ii) power coupling from the transmitted modes to their neighboring modes, resulting in crosstalk between OAM channels. Since the interface effects are dynamic, the link would experience time-varying degradations. We note that the beam could be outside the receiver aperture due to beam wandering and thus may not be efficiently captured without beam tracking. Therefore, to investigate the modal coupling and power loss caused by other distortions, we use a tracking system to partially mitigate the beam wandering for most of the measurements excluding Figure 3c.

**Figure 2:**
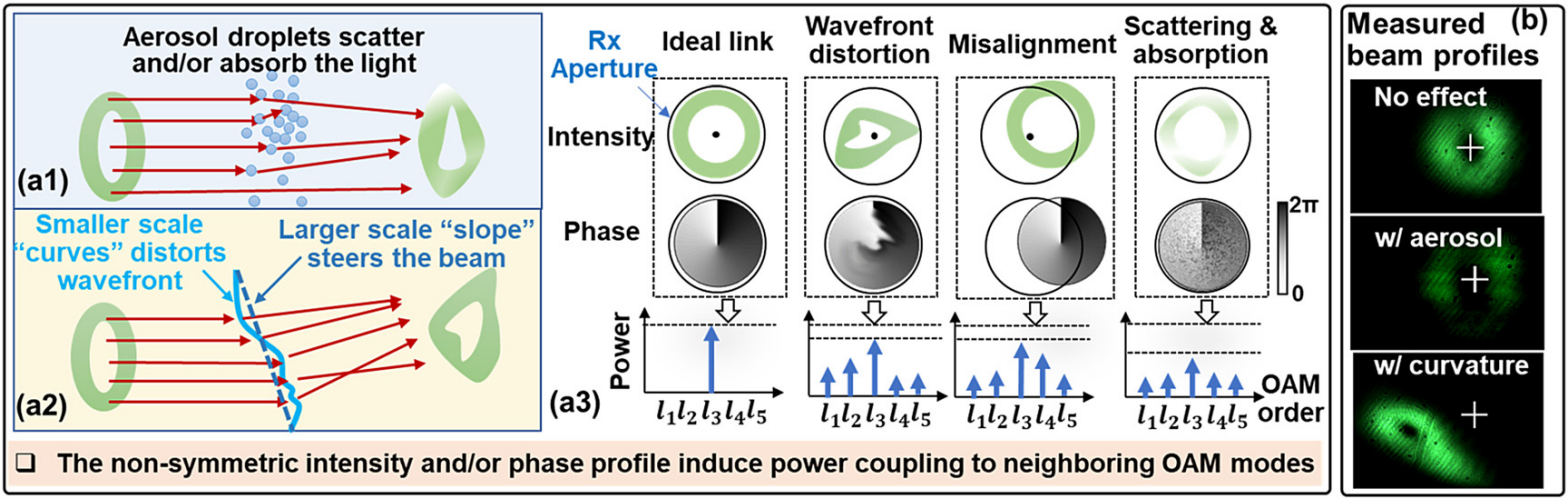
(a) The potential causes of modal power loss and power coupling under (a1) aerosol and (a2) curvature effects. (a3) The wavefront distortion, beam wandering, and spatially dependent scattering & absorption could affect the unique intensity and phase profile of the received OAM beam. (b) The example images of OAM −1 under aerosol and curvature effects. The cross mark in the image shows the beam center position under the no-effect case.

Figure 2a shows the potential causes for the power loss of the transmitted mode and the power coupling to neighboring modes under the dynamic aerosol and water surface curvature effects. As shown in Figure 2a1, the aerosol droplets could scatter and/or absorb the incident light [12], and the inhomogeneous aerosol could induce spatially dependent scattering and attenuation. In this case, the intensity and phase profile of the received beam could be affected, resulting in modal power coupling [25]. As shown in Figure 2a2, non-uniform water surface curvature could induce spatially dependent refraction toward the OAM beam. Specifically, there could be a relatively larger scale “slope” that steers the beam and induce beam wandering [14], and there could also be “curves” that are smaller than or similar to the beam size and induce wavefront distortion [15]. Both the beam wandering and wavefront distortion could affect the unique intensity and phase profiles of the received OAM beam, resulting in modal power coupling, as shown in Figure 2a3. We note that both types of water curvature structure may exist in our experiment. Several images of OAM −1 under aerosol and curvature effects are shown in Figure 2b as examples.

**Figure 3:**
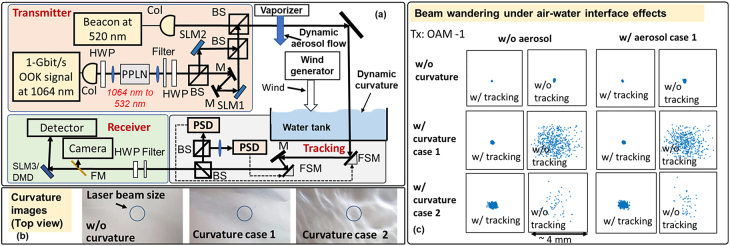
(a) The experimental setup for the OAM multiplexed link through the dynamic air–water interface. The aerosol flow is generated by a vaporizer, and the curvature of the interface is induced by wind. Col, collimator; PPLN, periodically poled lithium niobate; BS, beam splitter; SLM, spatial light modulator; FSM, fast steering mirror; PSD, position-sensitive detector; HWP, half-wave plate; DMD, digital micromirror device. (b) Example images of the curvature at the air–water interface. (c) The positions of the beam center (blue dots) over ∼1 min under various air–water interface effects with and without tracking. Under aerosol case 2, the received power is too low to be measured by the camera. Moreover, the beam is out of the camera for most of the measurements under curvature case 2 without tracking, and therefore there are fewer data points.

## 3 Experimental setup

In this experiment, we induce the aerosol and curvature of the air–water interface in two separated containers. The dynamic aerosol flow is composed of water droplets generated by a vaporizer, and we characterize the aerosol effect by its attenuation [36]. Specifically, aerosol cases 1 and 2 have attenuation coefficients of ∼0.1–0.6 and ∼0.7–1.3 dB/cm, respectively, over a distance of ∼7 cm. Such aerosol might have less thickness and a larger attenuation coefficient as compared with the ocean aerosol (several dB/km over several kilometers [36]) due to the limitation of our laboratory environment. The dynamic air–water interface curvature is induced using a wind generator. To characterize the curvature effects, we (i) measure the beam center offset of the refracted beam over ∼1 min and calculate the slope values (*φ* rad) at each moment [11], and (ii) calculate the variation of the curvature slope *φ* over time. Curvature cases 1 and 2 have slope variances of ∼6 × 10^−7^ and ∼2 × 10^−5^ rad^2^, respectively, and their maximum slopes are ∼0.003 and ∼0.015 rad, respectively. Such water curvature has a height of up to several millimeters. We note that the curvature slope could be more than 0.1 rad with a variance of >3 × 10^−3^ rad^2^ in the ocean [37], which is stronger than that of our experiment. The potential approaches to enable OAM transmission in stronger curvature will be discussed in Section 6. Moreover, as shown in Figure 3b, the “structure” of the curvature (i.e., the brightness variation recorded by the camera) could have a similar size to the transmitted beam and potentially result in wavefront distortion besides the beam wandering. Additionally, the aerosol and curvature perturbations reside in separated containers, so we can independently control both effects. However, there will be divergence of the beam during its propagation between the two containers, which may affect the degradation of the beam.

The experimental setup is shown in Figure 3a. At the transmitter, a 1-Gbit/s OOK signal is modulated on a laser at 1064 nm, and light is coupled into free space through a collimator. Subsequently, the data-carrying beam is coupled into a PPLN for frequency doubling. Through second harmonic generation (SHG), the 1064 nm beam is converted to a 532 nm wavelength Gaussian beam (green light) with a beam diameter of ∼3 mm. The resulting beam is split into two branches by a BS, and one branch is delayed in free space to decorrelate the two data channels. In each branch, an SLM converts the incoming Gaussian beam into an OAM beam with a unique order. The two OAM beams are subsequently multiplexed using a BS and combined with the beacon Gaussian beam (i.e., the beam for tracking) at 520 nm. The combined beams are transmitted through the aerosol and curvature containers and processed by a tracking system (response speed ∼1 kHz, and steering range ∼±0.026 rad). Finally, the OAM beam with the desired order is down-converted to a Gaussian beam by a DMD or an SLM and coupled into a single-mode fiber for analysis. Figure 3c shows the beam center (i.e., the blue dots in the figure) variation over ∼1 min recorded by a camera. The results show that the interface effects could induce beam wandering and such an effect is partially mitigated by the tracking system. Therefore, the measured degradations of the OAM beams are always affected by the residual misalignment. Such residual misalignment is relatively large under curvature case 2, which might be due to the limited tracking accuracy. Moreover, the power of the received beam is too low to be measured by the camera under aerosol case 2.

We note that the data-carrying beam at 532 nm is typically generated by (i) directly modulating the driving current of a blue-green laser diode (LD) or (ii) using an external modulator to modulate the light from the LD [4]. However, such approaches generally have a limited bandwidth of several GHz [4]. One approach that could potentially generate higher-bandwidth and higher-data rate signals at 532 nm is to use SHG. One report shows that a 10-Gbit/s OOK signal at 532 nm can be generated by modulating the signal on a 1064 nm laser using a commercial high-speed modulator and converting it to 532 nm through SHG [38]. In our experiment, we transmit a 1-Gbit/s OOK signal per channel as our bandwidth is limited by (a) the bandwidth of our receiver (∼1.6 GHz), and (b) the total transmitter power (as the SNR at the receiver is not adequate to efficiently recover higher-bandwidth signals).

Moreover, we recover one OAM beam at a time by converting it back to a Gaussian beam using SLM and then couple the Gaussian beam into a single-mode-fiber-based receiver. To extend our scheme for demultiplexing multiple OAM channels simultaneously, one may need to (i) use multiple BSs to split the incoming beams into multiple copies at the receiver and (ii) detect different OAM channels from different copies. It could be challenging for demultiplexing a large number of OAM channels by using such a scheme due to the loss induced by BSs. To reduce such demultiplexing loss, one may use multi-plane-light-conversion-based OAM demultiplexers instead [39].

## 4 Air–water interface effects for OAM beams

**Figure 4:**
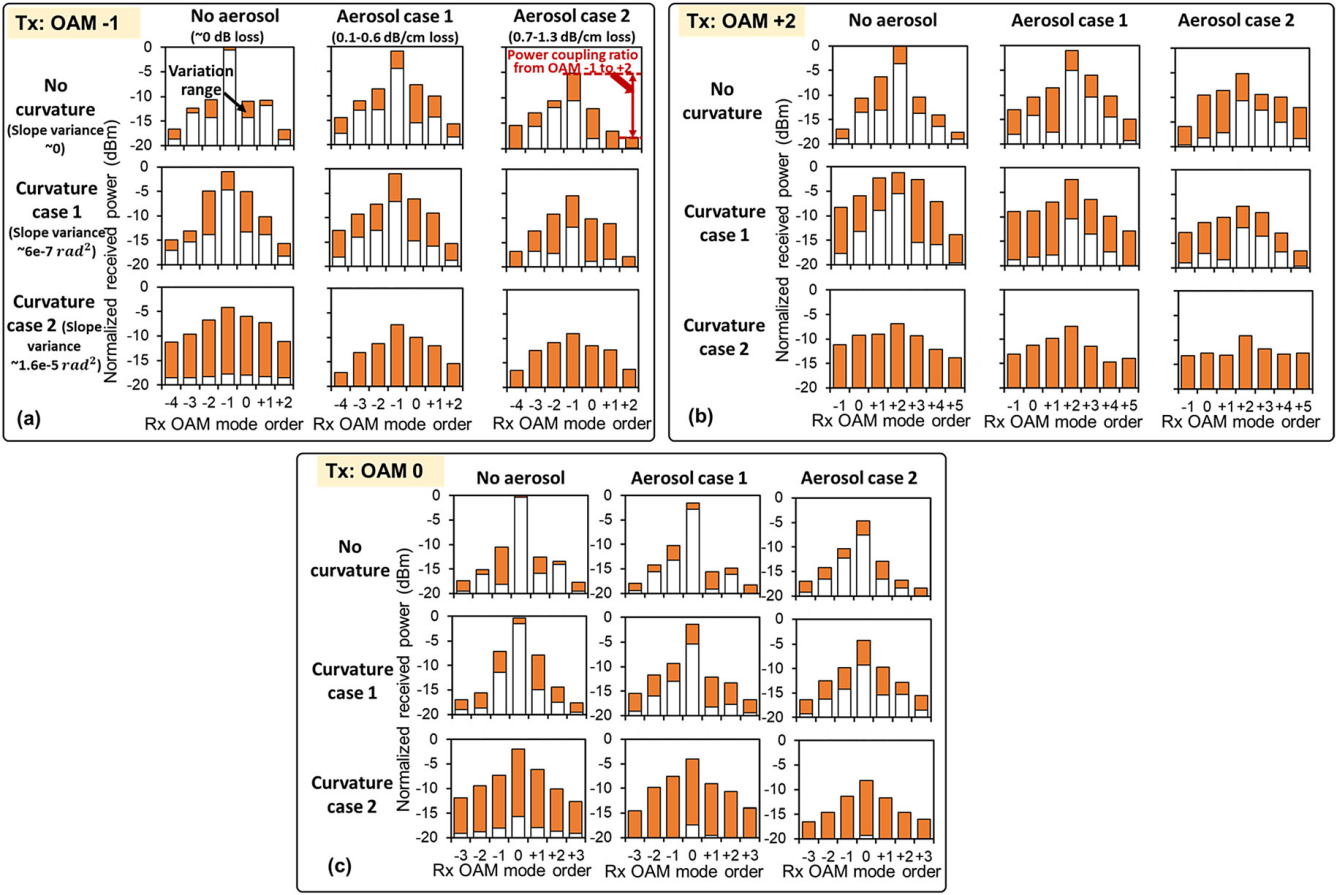
The received power on the transmitted modes and their neighboring modes under various aerosol and curvature conditions for (a) OAM −1, (b) OAM +2, and (c) OAM 0 (Gaussian beam). The beam propagation distance in aerosol is ∼7 cm. The orange bar shows the received power fluctuation range during the multiple measurements over >5 s, and each measurement takes ∼11 ms. The incident angle is 0 (normal incidence). The power is normalized by the maximum received power for the transmitted mode in the absence of interface effects.

Figure 4 shows the power loss of different transmitted modes and the power coupling to their neighboring modes under various aerosol and curvature conditions. In this measurement, we (i) transmit one beam at a time (OAM −1, +2, or 0), (ii) quickly change the mode demultiplexing pattern (each for extracting one OAM mode) on the DMD at the receiver at a rate of ∼1 kHz such that the received power on different time slot represents the power coupled to different modes, (iii) repeat this measurement multiple times and record the power variation over time using an oscilloscope, and (iv) calculate the power fluctuation range for each OAM mode (plotted as the orange bar in Figure 4). The DMD was used in this modal spectrum measurement because its pattern-switching rate is higher than that of our SLMs (∼10 Hz). In this section, we investigate the modal power loss and power coupling of OAM beams to show the potential causes for link degradations (OAM −1 and +2 multiplexed link). In this experiment, we multiplex OAM −1 & +2 instead of using OAM +1 & +2 or OAM −1 & +1 due to the lower inter-channel crosstalk between OAM −1 and +2. This is because a larger modal channel spacing (i.e., the difference between their OAM orders) could generally lead to lower channel crosstalk [24, 31]. The modal power loss and power coupling of a Gaussian beam, which is used in conventional FSO links, is also investigated for comparison with that of the OAM beams.

As shown in Figure 4, aerosol cases 1 and 2 could reduce the maximum received power on the transmitted mode by >1 and >5 dB, respectively. Moreover, as compared with the no aerosol case, the mutual power coupling ratio (defined as power on the received mode divided by power on the transmitted mode) between OAM −1 and OAM +2 increase by 2 and 5 dB under aerosol case 1, respectively, and increase by 4 and 7 dB under aerosol case 2, respectively. The reason could be that the inhomogeneous absorption and scattering of aerosol induce amplitude and phase distortion to the transmitted beams [25]. The curvature effects could also degrade the transmitted OAM beam. Specifically, the modal power loss for the transmitted OAM −1 and OAM +2 is >1 dB under curvature case 1, and >4 dB under curvature case 2. Moreover, the mutual power coupling ratio between OAM −1 and +2 increases by 11 and 13 dB under curvature case 2, respectively, as compared with the no curvature case. This is due to: (i) the curvature induces wavefront distortion of the transmitted beams [15], and (ii) there is residual misalignment between the incident beam and the receiver due to the beam wandering, as shown in Figure 3c. Both the wavefront distortion and misalignment could affect the amplitude and phase profile of the beam, and induce both power loss of the transmitted mode and power coupling to its neighboring mode. With the comprehensive effect of aerosol and curvature, there is a higher modal power coupling ratio and/or a higher modal power loss as compared with the single-effect cases. In our experiment, the curvature effect induces stronger beam degradation as compared with the aerosol effect, which might be due to that curvature effect induces larger beam wandering and thereby larger residual misalignment as compared with that of the aerosol effect, as shown in Figure 3c. We also show the modal power coupling of a Gaussian beam in Figure 4c for comparison. The Gaussian beam experiences lower power loss on the transmitted mode and has a lower modal power coupling ratio to neighboring modes under curvature case 1 and/or aerosol case 1 compared with OAM −1 and +2. This might be due to the smaller size of the Gaussian beam compared to that of OAM −1 and +2 [19], and thus the Gaussian beam experiences less amplitude & phase variation across the beam.

**Figure 5:**
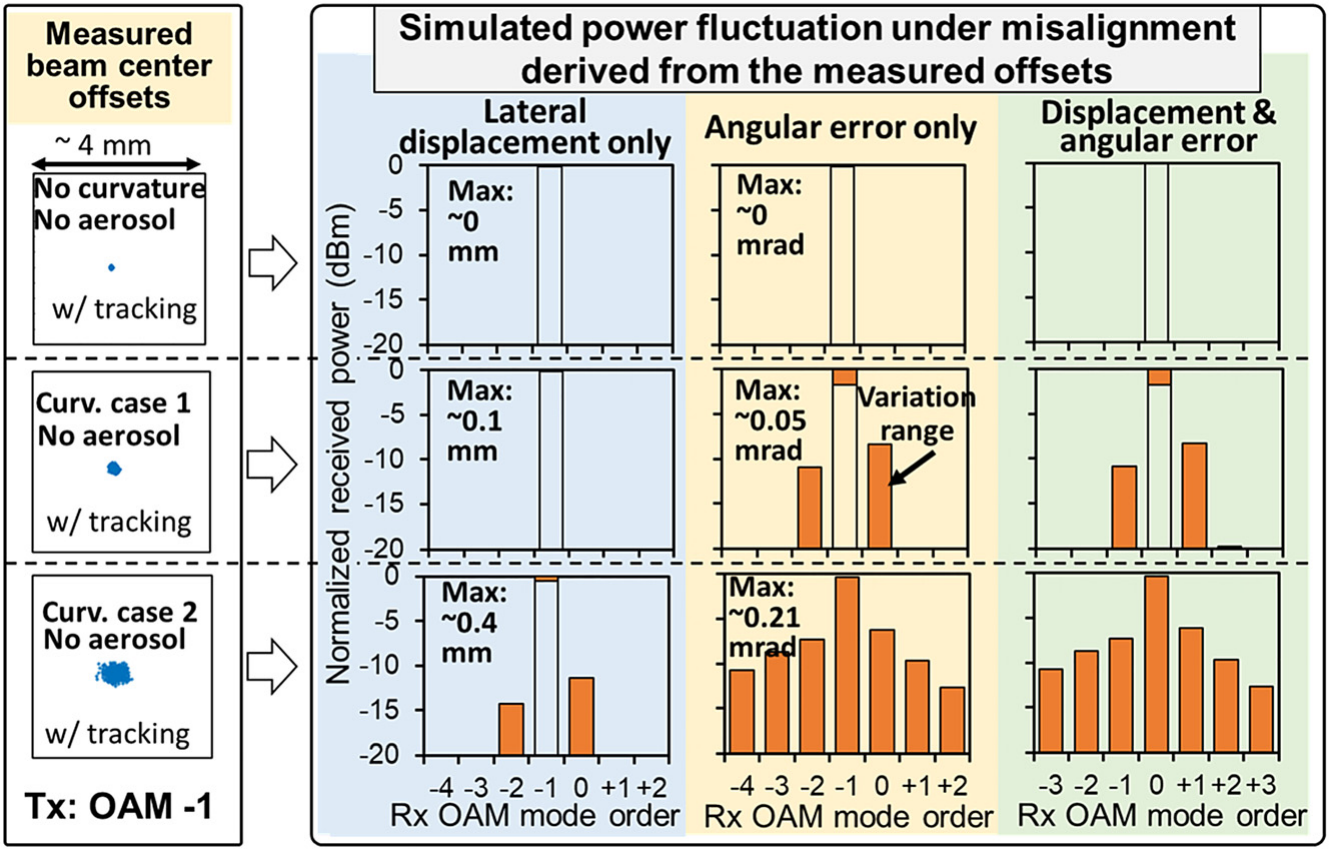
Simulated received power on each mode when OAM −1 is transmitted under the lateral displacement, angular error, and pointing error (both lateral displacement and angular error). In this simulation, we assume there is only beam misalignment without any other beam distortions. The beam diameter is ∼3 mm and the wavelength is 532 nm.

To further investigate the potential causes of modal power loss & modal coupling and to provide some insights into the experimental data, we simulate the power fluctuation on each mode for the transmitted OAM −1 under beam wandering effects, as shown in Figure 5. The misalignment values used for this simulation are derived from the measured beam offsets under three different example curvature conditions. Moreover, we assume the offset is caused by the pointing error without other distortions. Such pointing error includes both lateral displacement and angular error between the receiver and the beam [40]. We calculate the misalignment values as the following: (i) the lateral displacement is calculated as the measured beam center offset, (ii) the angular error is calculated as lateral displacement divided by the propagation distance from the air–water interface to the receiver (∼2 m), and (iii) the pointing error includes both lateral displacement and angular error. Subsequently, we simulate the power fluctuation on each mode under the lateral displacement, the angular error, and the pointing error, and the power is normalized by the transmitted power. The simulation results show that if there is only lateral displacement, there could be a modal coupling ratio of up to −8 dB, and if the angular error exists, there could be up to −6 dB modal coupling ratio and >20 dB power loss of transmitted mode. This is because the received beam’s phase and amplitude profiles are no longer azimuthally symmetric under misalignment [40]. Such misalignment might contribute to the beam degradations measured in Figure 4a–c. For our measurement, the angular error induces larger crosstalk as compared with the lateral displacement, which could be due to the specific values we use under the assumption that the offset is caused by the pointing error. The results could be different with the further increase of the lateral displacement.

**Figure 6:**
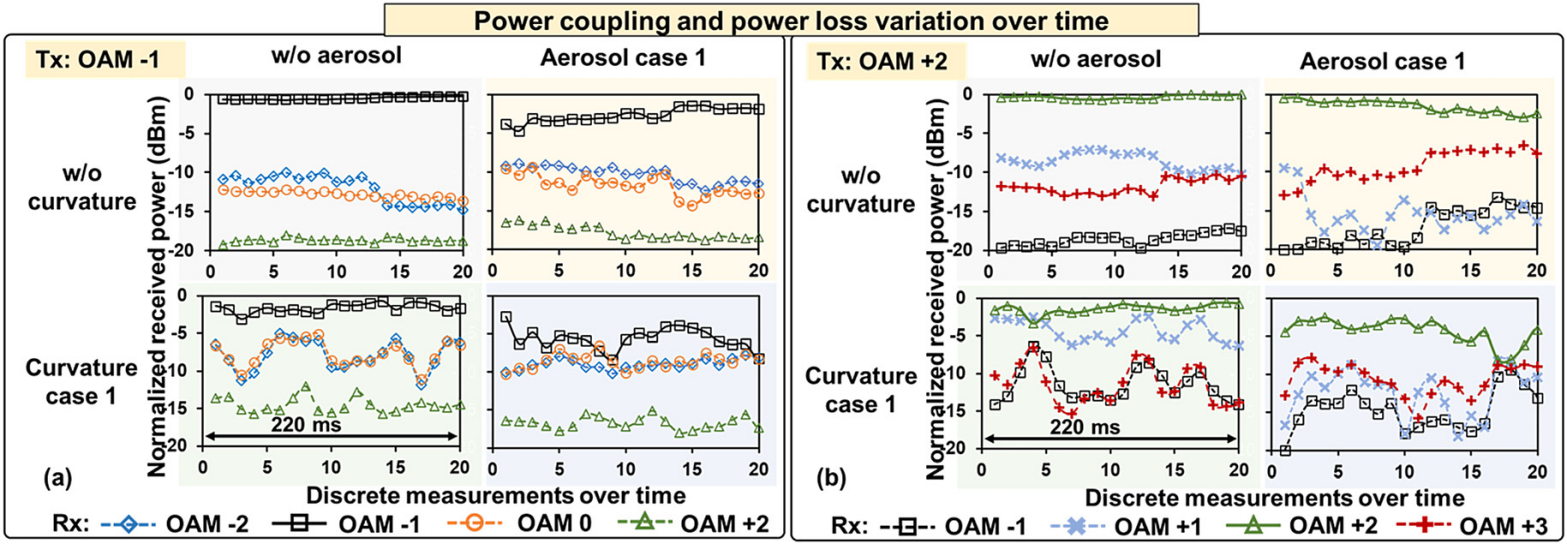
The power on each mode when (a) OAM −1 and (b) OAM +2 are transmitted. Each measurement takes ∼11 ms. The aerosol and curvature conditions with the same measurement number are different for different scenarios and different transmitted modes, and therefore they can’t be compared directly. The incident angle is 0 (normal incidence). The power is normalized by the maximum received power for the transmitted mode in the absence of interface effects.

Figure 6 shows the power loss of the transmitted mode and power coupled to neighboring modes over time. Each measurement takes ∼11 ms and there could be a small interval between subsequent measurements. The modal power loss and power coupling in each discrete measurement are measured by switching the demultiplexing pattern on the DMD (similar to the measurement for Figure 4). The results show that the crosstalk and power loss vary over time within the ∼220 ms measurement time. This could be because (i) the dynamic effects induce time-varying amplitude and phase distortion [15], and (ii) the residual misalignment varies with time due to beam wandering [40]. We note that we take the measurements under different interface effects sequentially and transmit one OAM mode at a time. Due to the dynamic nature of the aerosol and curvature, their conditions for data points with the same measurement number are not the same for different scenarios and different transmitted modes.

**Figure 7:**
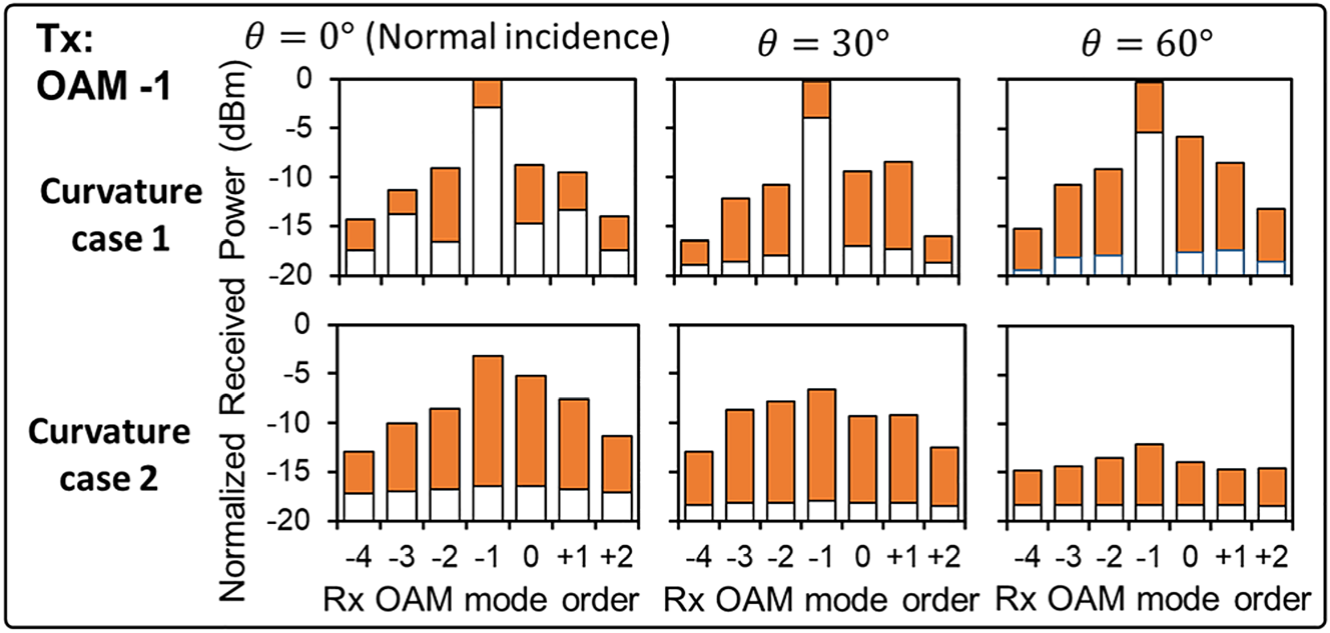
The received power on the transmitted mode and power coupling to the neighboring modes under different angles of incidence. The orange bar shows the received power fluctuation range for each mode. The power is normalized by the maximum received power for the transmitted mode without curvature. There are no aerosol effects for these measurements.

Figure 7 shows the power loss of the transmitted OAM −1 and power coupling to its neighboring modes under various angles of incidence. Under curvature case 1, the power loss of OAM −1 increases by up to 3 dB, and the modal power coupling ratio to neighboring modes increases by up to 3 dB as the angle of incidence increases. This might be because under a larger angle of incidence (i) the curvature induces stronger amplitude and phase distortion to the beam [41], and (ii) the curvature-induced beam wandering becomes stronger [42] and thereby result in a larger residual misalignment. We note that there is no aerosol effect for the measurements in Figure 7. Moreover, we rebuilt the tracking system and receiver for measuring the data in Figure 7 which may make it is difficult to compare the results in Figure 7 to those in Figures 5 and 6 directly.

## 5 Air–water interface effects for OAM multiplexed links

**Figure 8:**
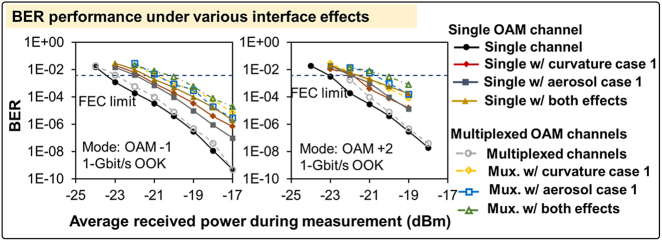
The BER performance of the OAM −1 and +2 under various interface effects. The measurement of each data point takes >30 s. We note that the interface conditions could be slightly different for all these measurements due to the dynamics of the aerosol and curvature. The incident angle is 0 (normal incidence).

Figure 8 shows the BER performance versus the average received power when a single OAM −1, or a single OAM +2, or multiplexed OAM −1 and +2 beams are transmitted. When only a single data channel is transmitted, the link experiences a ∼2 dB power penalty for the BER at the 7% FEC limit under comprehensive effect of aerosol case 1 and curvature case 1, when compared with the no-effect case. This is because the dynamic aerosol and curvature induce time-varying power loss of the transmitted mode, and there could be some time slots where the received power is too low to efficiently recover the signal. When OAM −1 and +2 are multiplexed, the link suffers from an extra ∼1 dB power penalty for the BER at the 7% FEC limit compared with the single-channel link. This is because the crosstalk between the two channels further degrades the link. We note that due to the OAM +2 channel having a higher loss than OAM −1 channel, its maximum received power is lower and fails to achieve a BER <1 × 10^−9^. Moreover, we use an SLM instead of a DMD to demultiplex the incoming OAM beams for BER measurements, because the DMD would induce a higher power loss (>10 dB) and degrade our link performance [43].

**Figure 9:**
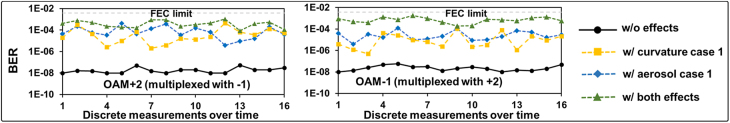
The BER performance for discrete measurements (each takes ∼4 s) over time under various air–water interface effects when OAM −1 and +2 are multiplexed. The four BER curves in each plot are not measured at the same time, and therefore should not be compared with each other directly. The incident angle is 0 (normal incidence).

Figure 9 shows the BER performance over time for the multiplexed OAM −1 and OAM +2 channels. Each of the BER measurements takes ∼4 s and the received power fluctuates around −18 dBm during the measurement. The BER performance for both channels varies with time, which is due to (i) the dynamic power loss results in a dynamic signal-to-noise ratio at the receiver, and (ii) the dynamic modal power coupling leads to time-dependent channel crosstalk. Here, we take the measurements for different interface conditions separately and only one of the two channels is recovered at a time. Therefore, the different BER measurements with the same measurement number only indicate the BER fluctuation trends and should not be compared directly. We note that due to the measurement for each BER point takes ∼4 s, the aerosol and curvature dynamics within a smaller time scale might be missed.

## 6 Summary and discussion

We experimentally investigate the effects of the dynamic aerosol and water surface curvature on the propagation of OAM beams. The results show that both the aerosol and curvature induce dynamic beam distortions, resulting in the power loss of the transmitted beam as well as the power coupling to neighboring modes. We also demonstrate a 2-Gbit/s OOK OAM-multiplexed link through aerosol and curvature effects. With some power penalty as compared with the no effects case, the OAM multiplexed link is achieved under aerosol case 1 and curvature case 1. To enable data transmission under stronger aerosol and/or curvature cases, we might need to (i) transmit higher power for the data channels, (ii) improve the accuracy and steering range of the tracking system to mitigate the residual misalignment shown in Figure 3c, and (iii) adaptively compensate for the amplitude and phase distortion, e.g., by using adaptive optics [16]. Moreover, we investigate the aerosol and curvature effects by varying the attenuation coefficient and the variance of the curvature slope, respectively. Other parameters (e.g., size of the aerosol particles and temporal frequency spectrum of the curvature) could also be varied to further investigate the air–water interface effects [13, 44].

In this experiment, we only multiplex two OAM beams for data transmission as a proof of concept due to limited transmitter power. It is likely possible to multiplex more than two OAM channels if higher transmitter power is used (e.g., by using a YDFA with higher output power). When more modal channels are transmitted, the performance of the center and side channels (at the center or on the side in terms of their OAM orders) may depend on their modal spacings and OAM orders. Specifically, (i) the modal channel crosstalk is generally lower when there is a larger modal spacing between different channels (the difference of their OAM orders) [24]; (ii) channels with higher OAM orders have larger beam sizes [19] and thereby may suffer more severe distortions and higher modal power loss under environmental effects as compared with the lower-order ones [45]. We note that the side channels (on one side or both sides) may have higher OAM orders as compared with the center channels. To optimize the link performance, one may need to consider both effects: the modal spacing between center and side channels should be large enough to reduce channel crosstalk, but also not too large such that the side channels could have relatively low OAM orders and experience relatively weak distortions [45].
